# Degenerative Scoliosis Correction Is Safe in Elderly Patients with Coronary Artery Disease

**DOI:** 10.3390/jcm15020729

**Published:** 2026-01-16

**Authors:** Yousaf B. Ilyas, Mojeed Fagbemi, Kristina P. Kurker, Gabriel S. Gonzales-Portillo, Dario A. Marotta, Morteza Sadeh, Nauman S. Chaudhry, Ankit I. Mehta

**Affiliations:** 1 University of Illinois College of Medicine, Chicago, IL 60612, USA; 2 Chicago Medical School, North Chicago, IL 60064, USA; 3 Department of Neurosurgery, University of Illinois, Chicago, IL 60612, USA; 4 Orlando Health Neuroscience Institute, Orlando, FL 32806, USA; nauman.chaudhry@orlandohealth.com

**Keywords:** scoliosis, coronary artery disease, deformity, degenerative

## Abstract

**Background/Objectives:** Coronary Artery Disease (CAD) is one of the leading causes of death in the United States. Although there is a plethora of studies about CAD, there remains a gap in the literature in examining the role of CAD in patients who undergo spine surgery. In this study, we examine the role of CAD in postoperative outcomes in adult patients who underwent surgery for degenerative scoliosis. **Methods:** The Scoliosis Research Society Database was queried for patients with degenerative scoliosis and divided into two cohorts: CAD and non-CAD. To minimize confounding bias, propensity score matching was done on comorbidities and patient demographics. Outcomes examined included: intraoperative complications, postoperative outcomes, and mortality rate. After matching, there were 139 patients in each group. **Results:** The CAD group had significantly higher rates of cardiac-related complications (5.8% vs. 0%, *p* = 0.012). No other intraoperative complications had significant differences between the groups. Interestingly, the non-CAD group had both a higher rate of returning to surgery (46.8% vs. 33.8%, *p* = 0.038) and antibiotic-related complications (5.8% vs. 0.7%, *p* = 0.042) respectively. There were no other differences regarding postoperative outcomes, including mortality. **Conclusions:** Our study found that aside from cardiac-related complications, the CAD group did not have any worse outcomes, and in some cases did better. These results are promising and may be due to more extensive preoperative screening and more risk aversion in patients with CAD. Our findings suggest that if spine surgeons exercise risk management for cardiac complications, CAD patients may benefit greatly from scoliosis surgery at no increased risk.

## 1. Introduction

Of the various spinal pathologies, scoliosis has become extremely common in the elderly population, with more than ⅓ one-third of people over 60 years old suffering from scoliosis [1]. Degenerative scoliosis in the elderly is associated with significant pain, neurological symptoms, functional limitation, and reduced quality of life; surgical correction has been associated with robust improvements in these domains even when postoperative complications occur [2,3]. Elderly populations are also well-documented to present with higher rates of various comorbidities. Coronary artery disease (CAD) is an especially prominent comorbidity, with the Centers for Disease Control and Prevention finding more than 14% of elderly patients having CAD [4]. In addition to its prevalence, CAD is particularly virulent, with some studies finding it to be the leading cause of death worldwide [5,6]. Within the surgical setting, CAD magnifies the physiological stress and is associated with higher rates of perioperative complications and mortality [7,8].

In addition to worse outcomes, patients with CAD have also been shown to be at higher risk for perioperative cardiovascular complications [9]. Current risk stratification for these patients relies on dividing them into different risk categories, with high-risk patients benefitting from intraoperative methods such as beta-blockers and antiplatelet therapy, while lower-risk patients receive preoperative stress testing [10,11,12]. Furthermore, tools such as the Revised Cardiac Risk Index (RCRI) have been developed, which quantify the risk of adverse cardiac events during and following surgery [13].

While the literature has found general trends for CAD patients undergoing surgery, there are still several gaps that exist that need to be explored further, especially within spine surgery. The RCRI has not been validated in spine surgery, with Carabini et al. finding that it was no better than chance when predicting cardiac morbidity postoperatively, thus indicating it cannot be used as a risk-stratification tool within spine surgery [14]. Given that scoliosis surgery offers meaningful functional and quality-of-life benefits to older adults, accurately identifying which CAD patients can safely undergo correction versus those who should defer surgery is critical for optimizing patient selection and perioperative decision-making [15]. Most existing studies examining cardiac history and outcomes in spinal fusion have focused on broad trends, showing that a history of cardiovascular disease is associated with poorer postoperative outcomes [16,17]. However, these analyses fail to isolate specific spinal pathologies, limiting the generalizability of their findings. This lack of distinction represents a significant gap in understanding how different spinal diseases uniquely interact with cardiovascular health. To date, no study has specifically evaluated how coronary artery disease influences perioperative complications and surgical safety in degenerative scoliosis correction, leaving surgeons without pathology-specific cardiac risk data to guide patient counseling and operative planning.

The inclusion of granular detail, such as spinal pathology, would offer valuable data for surgeons to practice more precise perioperative risk management and patient selection for elective spinal cases. Accordingly, our study seeks to be the first to analyze the role CAD has in scoliosis surgery by examining intraoperative and postoperative outcomes compared to a non-CAD group.

## 2. Materials and Methods

### 2.1. Study Population

The Scoliosis Research Society (SRS) Database is a national registry that contains deidentified data for more than 5000 patients who have undergone scoliosis correction surgery in the United States. The database contains the age, complications, and comorbidities of patients. We first identified 1313 patients with degenerative scoliosis from 2013–2023. As a de-identified database, we received an institutional review board (IRB) exemption when utilizing this dataset.

### 2.2. Data Source

A retrospective cohort study was conducted using data from the SRS database, a multi-institutional registry that prospectively collects detailed clinical, surgical, and outcomes data on patients undergoing operative management for scoliosis. From this database, we first identified all patients with complete records for primary curve magnitude and surgical intervention. Patients were then categorized based on the four scoliosis subtypes: idiopathic, degenerative, congenital, and neuromuscular.

### 2.3. Variables and Outcomes Collected

Comorbidities were extracted, including alcohol abuse, anemia, cancer, Collagen Vascular Disease, Diabetes Mellitus, hypertension, heart disease, kidney disease, liver disease, neurologic disease, obesity, osteopenia, and smoking status. Age of patients and levels fused were also extracted. Complications examined include: cardiac-related complications, pulmonary embolism, sepsis, respiratory-related complications, mortality (within 90 days of surgery), new postoperative neurologic deficit, return to surgery, loss of correction, change in neurologic status, complications from antibiotics, instrumentation reinserted, instrumentation removed, post-operative wound infection, and duration of post-operative wound infection (days).

### 2.4. Propensity Score Matching

To minimize selection bias and ensure balanced comparison groups, we performed 1:1 nearest-neighbor propensity score matching (PSM) without replacement utilizing Python 3 on the CAD cohort with the non-CAD cohort. The matching algorithm utilized the following variables: alcohol abuse, anemia, cancer, collagen vascular disease, diabetes mellitus, hypertension, heart disease, kidney disease, liver disease, neurologic disease, obesity, osteopenia, smoking status, age (years), and levels fused (mean ± SD). Balance across matched groups was evaluated using standardized mean differences (SMDs), with an SMD < 0.15 considered acceptable.

### 2.5. Statistical Analysis

After 1:1 matching, 139 patients were included in each group, resulting in well-balanced cohorts with most variables demonstrating standardized mean differences (SMDs) < 0.15 (Table 1). The most prevalent comorbidities were heart disease (36% in each group) and hypertension (35.3% in the CAD group vs. 30.2% in the non-CAD group). Both cohorts had a mean age of 71 years.

**Figure 1:**
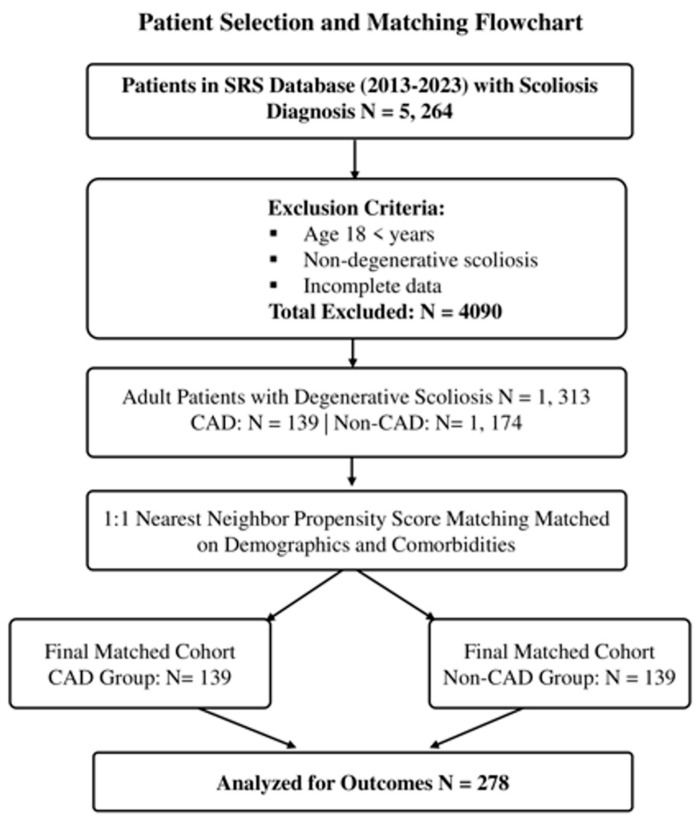
The Scoliosis Research Society (SRS) database was queried for adult patients (≥18 years) who underwent surgery for degenerative scoliosis between 2013 and 2023. Of 5264 total scoliosis cases, pediatric and non-degenerative scoliosis cases were excluded, leaving 1313 adult patients with degenerative scoliosis. Of these, 139 had coronary artery disease (CAD), and 1174 did not. After 1:1 propensity-score matching on demographics and comorbidities (nearest-neighbor, caliper 0.2), the final analytic cohort comprised 278 patients (139 CAD, 139 non-CAD).

In total, 278 matched patients were included in the final analysis (Figure 1). Independent *t*-tests and chi-square tests were used to compare differences between groups across the following variables: cardiac-related complication, pulmonary embolism, sepsis, respiratory-related complication, mortality rate (within 90 days of surgery), new postoperative neurologic deficit, return to surgery, loss of correction, change in neurologic status, complications from antibiotics, instruments reinserted, instrumentation removed, acute postoperative wound infection, and duration of acute postoperative wound infection (days).

## 3. Results

The CAD group had significantly higher rates of cardiac-related complications (5.8% vs. 0%, *p* = 0.012). No other intraoperative complications had significant differences between the groups. Interestingly, the non-CAD group had both a higher rate of returning to surgery (46.8% vs. 33.8%, *p* = 0.038), sepsis (4.3% vs. 0%, *p* = 0.039), and antibiotic-related complications (5.8% vs. 0.7%, *p* = 0.042), respectively. Despite the higher rate of antibiotic-related complications, there was no significant difference in the severity of these complications, as measured by the length of stay due to them. There were no other differences regarding postoperative outcomes, including mortality (see Table 2 for full results).

## 4. Discussion

This study investigated the impact of CAD on outcomes for elderly patients undergoing degenerative scoliosis correction surgery. We found that while patients with CAD experienced a significantly higher rate of postoperative cardiac-related complications, they did not have an increased risk of mortality. However, the non-CAD cohort had significantly higher rates of returning to surgery (46.8% vs. 33.8%, *p* = 0.038), sepsis (4.3% vs. 0.0%, *p* = 0.039), and antibiotic-related complications (5.8% vs. 0.7%, *p* = 0.042). Our results suggest that while CAD introduces perioperative risks, it does not appear to be an absolute contraindication for this elective degenerative scoliosis surgery, if patients are appropriately selected and managed.

Our finding that CAD increases the risk of cardiac events aligns with the broader spinal literature. A retrospective analysis of 1346 patients by Wang et al. identified a prior history of myocardial infarction (MI), hypertension, and atrial fibrillation as key predictors of 30-day perioperative MI after spine surgery [18]. Similarly, Bovonratwet et al. identified a history of cardiac disorders as a significant risk factor for perioperative cardiac arrest or myocardial infarction, increasing the relative risk by 1.88 [19]. Our study corroborates these previous findings, confirming that patients with CAD represent a higher-risk population for specific cardiac events during the perioperative period of spine surgery.

Our results show an absence of increased mortality in the CAD cohort, suggesting that cardiac risk, when anticipated, can be effectively mitigated. This is likely a direct consequence of the heightened perioperative vigilance applied to patients with known cardiovascular disease. In geriatric patients undergoing spine surgery, perioperative management literature emphasizes multidisciplinary risk stratification and individualized anesthetic planning, particularly in the presence of cardiovascular comorbidities [2]. This focused cardiac care creates a broader protective effect that prevents fatal complications. While the increased cardiac risk in the CAD cohort was an anticipated finding, the higher rates of reoperation, sepsis, and antibiotic-related complications in the non-CAD group were unexpected findings. One hypothesis is that the same intensive monitoring and risk-averse strategies applied to the CAD group offered a degree of protection against other non-cardiac adverse events.

These findings have important implications for surgical decision-making and patient selection in elderly spine surgery. Elective scoliosis surgery in the elderly requires rigorous patient selection, as advanced age and comorbidities are well-established risk factors for perioperative complications [2,3]. While heart disease is also a well-established risk factor in spine surgery, emerging evidence suggests that age and cardiac comorbidity no longer preclude safe operative intervention when appropriate precautions are taken [3,20]. Passias et al. demonstrated that despite rising surgical complexity and comorbidity burden in patients aged 75 and older, in-hospital complication rates declined from 26.7% to 8.6% between 2003 and 2012 [21]. Our finding of no increased mortality in the CAD cohort, despite higher cardiac complications, supports this trend and suggests that proactive cardiac management enables safe corrections of adult spinal deformity even in high-risk populations.

Our findings also help to define the specific risk profile of CAD, contributing to a more refined understanding of surgical risk in this patient population. While CAD increased cardiac complications, it did not increase mortality or reoperation risk. This is a critical distinction, as other cardiac conditions may confer different, and potentially more severe, risks. For instance, Ahmad et al. found that while patients with CAD had significantly increased odds of postoperative myocardial infarction, patients with congestive heart failure (CHF) demonstrated a broader pattern of risk, with higher rates of pneumonia, cerebrovascular accident, sepsis, and mortality after spinal fusion [22]. This concept extends to other commodities as well, with Soroceanu et al. finding that hypertension and smoking are independent predictors of medical complications but noted that patients who experienced these complications still achieved long-term functional improvements comparable to those who did not [23].

When compared to other comorbidities, CAD emerges as a key but not isolated risk factor for adverse outcomes in spine surgery. For example, atrial fibrillation—the most common clinically relevant dysrhythmia—has been associated with an increased risk of perioperative ischemic stroke in noncardiac surgical cohorts (e.g., adjusted OR ~2.1) and is linked with a markedly elevated long-term risk of thromboembolic stroke, particularly in older and high-risk patients [24,25]. Beyond cardiac conditions, diabetes, and chronic kidney disease independently increase the risk of perioperative complications and worse outcomes [26,27]. Importantly, although prior literature reports increased myocardial-infarction risk in CAD patients (odds ratio = 1.6), our findings of comparable mortality and reoperation rates between CAD and non-CAD cohorts suggest a more limited effect size than would be predicted from the broader comorbidity risk hierarchy [22]. For comparison, congestive heart failure carries a postoperative mortality odds ratio of 5.67, and pulmonary circulation disorders an odds ratio of 8.94 for mortality, underscoring that CAD’s perioperative threat is substantially more circumscribed and modifiable than many other conditions routinely encountered in spinal deformity correction [28]. Collectively, these comparisons support that while CAD warrants vigilant peri-operative cardiac management and optimized risk stratification, it should not prevent appropriately selected elderly patients from undergoing corrective scoliosis surgery.

In patients over 70 years old with degenerative lumbar scoliosis, Ding et al. found that lower BMI and a greater number of instrumented segments independently predicted perioperative complications, while the number of preoperative comorbidities and advanced age were not associated with higher complication rates [29]. They concluded that with appropriate perioperative management, acceptable perioperative outcomes can be achieved despite multiple comorbidities and advanced age. This risk profile highlights the need for spinal surgery literature to systematically report when comorbidities do not confer additional risk for key outcomes, such as mortality or major complications. Reporting this lack of findings is as important as reporting positive associations. Failure to do so may lead to an overestimation of risk and suboptimal surgical decision-making, particularly when counseling elderly patients for elective procedures.

Furthermore, older adults stand to benefit more from spinal surgery. Despite high complication rates (with over 70% of patients experiencing at least one complication), prospective multicenter data demonstrate significant 2-year postoperative improvements in pain, disability, and health-related quality of life, as measured by the Oswestry Disability Index (ODI), the Short Form–36 Health Survey (SF-36), and the Scoliosis Research Society–22 questionnaire (SRS-22)scores following adult spinal deformity surgery [30]. Notably, these long-term functional improvements were not significantly different between patients who experienced complications and those who did not, indicating that perioperative morbidity does not necessarily diminish the overall benefit of surgery [29]. Perhaps most compellingly, a risk-benefit analysis by Smith et al. concluded that elderly patients, despite facing the highest risk of perioperative complications, stand to gain a “disproportionately greater improvement in disability and pain with surgery” compared to younger cohorts [31]. This is because they often start from a lower functional baseline, with greater disability, worse health status, and more severe pain; thus, they have more potential for improvement.

Importantly, the long-term benefits of scoliosis surgery in the elderly extend beyond pain relief and deformity correction. Multiple studies demonstrate significant improvements in mobility and health-related quality of life [32,33]. This improved mobility may also confer secondary cardiovascular benefits, as increased physical activity is associated with reduced cardiovascular morbidity in older adults. In summary, the evidence supports a refined approach to patient selection for elective scoliosis surgery in the elderly, emphasizing comorbidity optimization and individualized risk assessment. The finding that stable CAD does not preclude safe surgical intervention, when managed appropriately, is consistent with current literature and underscores the potential for meaningful improvements in function and quality of life in this vulnerable population.

### 4.1. Strengths

The strengths of this study include its use of a large, national database, which enhances the generalizability of our findings. The matched cohort of 278 patients provides substantial statistical power for the detection of significant intergroup differences. Furthermore, the use of propensity score matching is a major methodological strength, as it allows us to create well-balanced cohorts and minimize the effect of confounding variables from patient demographics and comorbidities.

### 4.2. Limitations

This study has several important limitations that should be considered when interpreting the results. As a retrospective registry-based analysis, it is inherently susceptible to selection bias because patient cohorts cannot be randomized. Although propensity-score matching balanced major demographic and comorbidity factors, residual confounding likely persists. Patients with CAD who successfully proceeded to surgery are required to undergo stringent cardiology clearance and medical optimization; thus, those with more severe or unstable disease were likely screened out during pre-operative evaluation. This creates a cohort that is not fully representative of the broader CAD population and may underestimate perioperative risk, particularly in patients with advanced cardiovascular disease. Additionally, although the averages were comparable for levels fused, its SMD and the SMD for obesity were greater than 0.2, suggesting that the dataset was not completely balanced.

This study is also limited by its modest sample size, which reduced statistical power for detecting differences in rare but clinically important outcomes such as mortality, sepsis, and pulmonary embolism. Although the matched cohort included 278 patients, many postoperative complications occurred infrequently, preventing reliable multivariable modeling and increasing the likelihood of type II error. Despite these limitations, this study provides valuable early evidence on the relationship between CAD and outcomes in adult degenerative scoliosis surgery—a topic for which virtually no prior data exist. By using propensity score matching to minimize confounding, our analysis highlights several outcome differences that warrant further investigation and provides a foundation for larger prospective studies.

Furthermore, the granularity of cardiac disease was limited, as CAD was captured only as a binary variable. Important information, such as left-ventricular function, coronary lesion burden, timing and type of revascularization, and New York Heart Association functional classification, was unavailable. Patients with stable single-vessel stenosis may differ substantially in perioperative risk from those with multivessel disease or prior bypass grafting, yet such variability could not be analyzed, restricting the ability to perform refined cardiac risk stratification within the CAD cohort.

The SRS registry also lacks several domains critical for interpreting outcomes in adult spinal deformity surgery. Radiographic parameters, including curve magnitude, sagittal alignment, and pelvic parameters, were not captured, limiting the capacity to evaluate deformity severity or its contribution to postoperative risk. Similarly, intraoperative variables such as operative duration, blood loss, and specific instrumentation or osteotomy techniques were unavailable, reducing granularity in modeling risk and identifying mechanisms driving adverse events. Measures of frailty and nutritional status, which are established predictors of adverse events, extended hospital stay, and non-home discharge in elderly spine surgery patients, were not available, limiting our capacity to account for physiological vulnerability. Additionally, the absence of patient-reported outcome measures such as ODI, SRS-22, and PROMIS tools prevents assessment of whether comparable complication profiles between cohorts translate into similar improvements in pain, function, and quality of life.

Variability in documentation practices and perioperative management across participating centers introduces heterogeneity that may influence data completeness and external validity. Finally, as with all retrospective registry-based studies, causal inference cannot be established; while associations between CAD and postoperative outcomes can be identified, the data cannot prove that CAD directly caused those outcomes.

### 4.3. Future Directions

Prospective studies are needed to confirm these results and should incorporate PROs to assess the long-term, patient-centered value of scoliosis surgery in this population. Future analyses should also aim to investigate the effects of multiple, concurrent heart conditions to determine if specific combinations impart a synergistic risk greater than the sum of their individual parts. Finally, an intra-CAD analysis is needed to investigate whether a specific threshold of disease severity exists at which the risks of surgery begin to outweigh the benefits. Such research would provide surgeons with more precise data to guide the complex decision-making required for treating adult spinal deformity.

## 5. Conclusions

In conclusion, this propensity-matched cohort study demonstrates that a history of CAD is associated with a significantly increased risk of postoperative cardiac complications in elderly patients undergoing corrective surgery for degenerative scoliosis. However, this did not translate to an increased risk of mortality. Our findings suggest that while CAD necessitates careful preoperative evaluation and vigilant postoperative monitoring, it should not be considered an absolute contraindication for this procedure. With meticulous patient selection and tailored perioperative management, elderly patients with CAD can safely undergo major spinal deformity surgery to improve their quality of life.

## Figures and Tables

**Table 1:** Pre- and Post-Matching Demographic and Comorbidity Data.

Comorbidity	Pre-Matching Value CAD Group (N = 139)	Pre-Matching Value Non-CAD Group (N = 1174)	Pre-Matching SMD	Post-Matching Value CAD Group (N = 139)	Post-Matching Value Non-CAD Group (N = 139)	Post-Matching SMD
Alcohol Abuse	0.70%	1.00%	−0.033	0.70%	0.00%	0.12
Anemia	5.00%	1.90%	0.173	5.00%	5.00%	0
Cancer	2.20%	2.00%	0.014	2.20%	2.20%	0
Collagen Vascular Disease	0.00%	1.10%	−0.15	0.00%	1.40%	−0.17
Diabetes Mellitus	10.10%	8.30%	0.06	10.10%	8.60%	0.049
Hypertension	35.30%	19.20%	0.367	35.30%	30.20%	0.107
Heart Disease	36.00%	4.20%	0.862	36.00%	36.00%	0
Kidney Disease	4.30%	1.80%	0.147	4.30%	7.90%	−0.15
Liver Disease	0.70%	0.70%	0.005	0.70%	1.40%	−0.069
Neurologic Disease	2.90%	0.90%	0.15	2.90%	0.70%	0.162
Obesity	11.50%	8.30%	0.109	11.50%	5.80%	0.205
Osteopenia	12.20%	12.60%	−0.011	12.20%	11.50%	0.022
Smoking	5.80%	3.30%	0.117	5.80%	2.90%	0.141
Age (years)	71.1 (±) 7.0 years	67.7 (±) 8.4 years	0.45	71.1 (±) 7.0 years	70.9 (±) 5.8 years	0.029
Levels Fused (mean (±) SD)	8.1 (±) 3.4	8.3 (±) 4.2	−0.059	8.1 (±) 3.4	7.2 (±) 3.6	0.264

**Table 2:** Intraoperative and Postoperative Outcomes (*p*-values below 0.05 are bolded).

Outcome/Complication	CAD Cohort (%)	Non-CAD Cohort (%)	*p*-Value
Cardiac-Related Complication	5.755	0	**0.012**
Pulmonary Embolism	0.719	3.597	0.216
Sepsis	0	4.317	**0.039**
Respiratory-Related Complication	0.719	2.158	0.615
Mortality Rate	10.072	7.914	0.675
New Postoperative Neurologic Deficit	28.777	30.216	0.895
Return to Surgery	33.813	46.763	**0.038**
Loss of Correction	2.158	1.439	1
Change in Neurologic Status	0.719	2.878	0.367
Complications from Antibiotics	0.719	5.755	**0.042**
Instruments Reinserted	0.719	4.317	0.126
Instrumentation Removed	2.158	5.036	0.334
Acute Post-operative Wound Infection	0	0	1
Duration of Acute Post-operative Wound Infection (days)	53	71	0.413

## Data Availability

We utilized data from the Scoliosis Research Society Database, which is available for members.
